# University of Louisville

**Published:** 1853-02

**Authors:** 


### THE WESTERN JOURNAL.

Third Series.—Vol. XI.—No. II.

### LOUISVILLE, FEBRUARY 1, 1853.

### UNIVERSITY OF LOUISVILLE.

In our last number, our readers were presented with a history of the medical department of the University of Louisville, which we would feign flatter ourselves was not unacceptable to them. It may be presumed that the members of the profession feel an interest in the progress of the medical institutions of our country, by which the character of the profession is, to some extent, affected. They ought to be so far informed as to their condition and policy, as to be able when asked for an opinion to do justice to the several schools seeking their patronage. This is one reason for writing their history. Another is, that the persons connected with their origin are passing away, and the events of their earlier days are fading out of the memories of their cotemporaries and are likely to be lost unless put into some durable form. The founders of the University of Louisville believe, that it is an institution concerning the rise and progress of which there will be an interest felt by those who will come after them. We recur to the subject again for the purpose of saying that the catalogue of the medical class at present attending the course in the University is somewhat longer than the last—a circumstance which, we are sure, will give pleasure to many of our readers. For two years there was a declension in the size of its classes, and the cause which affected it injuriously at first having been repeated, last summer, it was feared that the current winter would exhibit a further decline; the resignation of two popular professors so shortly after occurrences of the same character was well calculated to bring about that result. That no such falling off has occurred is certainly a matter for gratulation with the friends of the school.

Dr. Flint and Dr. Palmer have proved to be all that their colleagues and pupils could desire in associates and teachers. More than this we could hardly say of them, and in justice to them we could not say less. As fortunate as the school has been in securing the services of able men to fill the vacancies which have occurred in it, it was never more fortunate than when it gained these gentlemen.

